# Editorial: Physical Activity: An Optimizer of the Neurophysiological System?

**DOI:** 10.3389/fpsyg.2021.754343

**Published:** 2021-09-17

**Authors:** Juan Pedro Fuentes-García, Rodrigo Ramirez-Campillo, Mauricio Garzón-Camelo, Maria António Castro

**Affiliations:** ^1^Didactic and Behavioral Analysis of Sports Research Group (ADICODE), Faculty of Sport Sciences, University of Extremadura, Cáceres, Spain; ^2^Department of Physical Activity Sciences, Universidad de Los Lagos, Osorno, Chile; ^3^Department of Physical Activity Science, Montreal University - University of Quebec, Montreal, QC, Canada; ^4^Higher School of Health, Polytechnic Institute of Leiria, Leiria, Portugal

**Keywords:** exercise, neurophysiology, nervous system, brain, executive functions, quality of life, aging, detraining

From the field of neurophysiology, broadly defined as the study of the nervous system function, numerous researches have studied the central and peripheral nervous systems from whole organs to subcellular compartments.

Different studies focused on physical activity and health have shown the impact of performing a physical fitness test on the simultaneous performance of a cognitive task, or the effects of exergames on heart rate variability in women with fibromyalgia (Villafaina et al., 2018, 2020). Other studies focused on cognitive performance have shown the psychophysiological stress response of adolescent chess players during problem-solving tasks (Fuentes-Garcia et al., 2019) or the electroencephalographic response of chess players in decision-making processes under time pressure (Villafaina et al., 2019).

In the same sense, different studies have shown that physical exercise improves the efficiency of the capillary system and increases the supply of oxygen to the brain, affecting the improvement of metabolic activity and oxygen intake in neurons (Kaliman et al., 2011). This positively influence different brain functions (e.g., attention) (de Bruin et al., 2016), and aerobic physical exercise may improve brain neurophysiological activity during the resolution of a selective attention test (Ferro et al., 2019) or working memory performance (Hsieh et al., 2018).

Consequently, there is evidence that participation in physical activity may modify white matter integrity and activation of regions key to cognitive processes. However, additional larger hypothesis-driven studies are needed to replicate findings (Valkenborghs et al., 2019). Physical activity buffers the negative effects of stress on cognitive performance in children (Wunsch et al., 2019), and may have a positive effect on memory, executive functions, and on genes associated with neuroprotective anti-aging resilience signaling (Corpas et al., 2019).

The main objective of this Research Topic was to gather studies that shed more light on the benefits of physical exercise in the neurophysiological system, from childhood to old age and from the field of health to sports or professional performance. For example, we consider important those studies that deepen into the epigenetic mechanisms involved in the aging process and their modulation through physical exercise, improving prevention and treatment therapies, and those that contributes to better understand how physical activity improves brain functions (e.g., increased hippocampal), or what effect cognitive loads cause in variables such as heart rate variability or brain waves. We also consider it particularly interesting to show studies that can reflect how physical exercise can be a good preventive strategy to avoid or counteract neurodegenerative diseases, such as Alzheimer, and consequently, increase the time and quality of life.

Thus, some of the topics of interest for this Research Topic are studies that contemplate the latest advances on neurophysiological and epigenetic effects of physical exercise on the aging, or beneficial effects of the practice of physical activity and sport on anti-aging and neuroprotective mechanisms. Equally relevant aspects to consider are the effects of physical exercise to prevent neurodegenerative diseases, the relationship between physical exercise practice and improvement of brain functions, the effects of cognitive loads at the neurophysiological level, or the neurophysiological system behavior related to sports or professional performance.

The influence of physical fitness in the nervous system structure is examined by Best et al. in siblings to estimate the contribution of genetic and environmental factors to variation within physical fitness and brain structure. Although performance-based measures of fitness were not associated with any structural neuroimaging markers, greater body mass index is associated with lower white matter integrity.

The effects of stress and lifestyle factors have been assessed with biomarkers like telomere length and telomerase activity which may have the potential to help the understanding of the stress-aging relationship and potential underlying mechanisms in elite athletes (Mehrsafar et al.).

The benefits of exercise are documented for different diseases. Even though it is still to define the exercise intensity and frequency required to improve patients' autonomic modulation, positive effects provided by exercise programs enhanced by resistance and endurance are found in cancer patients and survivors as demonstrated by Lavín-Pérez et al..

The study performed by Chi-Fang Lin et al. suggests that in children with Attention-Deficit/Hyperactivity Disorder Theta/Beta Ratios may be one of the mechanisms between motor ability and inhibition function.

Still concerning pathological conditions, Villafaina et al. explained that the performance during motor–cognitive dual and single tasks in women with fibromyalgia show the same electrical brain activity pattern whereas healthy ones adapt brain activity to task commitment.

Among heroin addicts, Wang et al. demonstrated that aerobic exercise attenuates heroin cravings and promotes inhibitory control showing its efficacy when dealing with this condition.

Exercise has been shown to optimize older adults sleep, especially, when moderate-intensity continuous training and stretching are considered, showing greater efficacy than high-intensity interval training (Bullock et al.). A similar effect is found concerning stress in different age populations confirming the health-enhancing effect of acute exercise (Mücke et al.) and physical activity amount on some of the physiological and psychological stress reactivity indicators as well as the central fatigue and perceived exertion (de la Vega et al.).

The neuroprotective effect of exercise in the aging process has been explored by Burgos et al. Executive function in normally aging adults has shown to beneficiate from the practice of exercise. An additional gain is exhibited when to the physical exercise, mental training is added, probably through more efficient early attentional processing.

This book explores some of the most UpToDate issues and raises questions to be answered by further research. Hopefully, this collection will stimulate this issue study.

## Author Contributions

JF-G, RR-C, MG-C, and MC conceived and designed the Research Topic and wrote the editorial. All authors contributed to the article and approved the submitted version.

## Conflict of Interest

The authors declare that the research was conducted in the absence of any commercial or financial relationships that could be construed as a potential conflict of interest.

## Publisher's Note

All claims expressed in this article are solely those of the authors and do not necessarily represent those of their affiliated organizations, or those of the publisher, the editors and the reviewers. Any product that may be evaluated in this article, or claim that may be made by its manufacturer, is not guaranteed or endorsed by the publisher.

## References

[B1] Corpas R. Solana E. De la Rosa A. Sarroca S. Grinan-Ferre C. Oriol M.. (2019). Peripheral maintenance of the axis SIRT1-SIRT3 at youth level may contribute to brain resilience in middle-aged amateur rugby players. Front. Aging Neurosci. 11:352. 10.3389/fnagi.2019.00352 31956305 PMC6951402

[B2] de Bruin E. I. van der Zwan J. E. Bogels S. M. (2016). A RCT comparing daily mindfulness meditations, biofeedback exercises, and daily physical exercise on attention control, executive functioning, mindful awareness, self-compassion, and worrying in stressed young adults. Mindfulness 7, 1182–1192. 10.1007/s12671-016-0561-5 27642375 PMC5010624

[B3] Ferro E. F. Cid F. M. Munoz H. D. Aburto B. N. Nogales O. G. Perez A. M. (2019). Effects of a session of physical exercise on the neurophysiological activity during the resolution of a test of selective attention. Retos-Nuevas Tendencias En Educacion Fisica Deporte Y Recreacion, 36, 391–397.

[B4] Fuentes-Garcia J. P. Pereira T. Castro M. A. Santos A. C. Villafaina S. (2019). Psychophysiological stress response of adolescent chess players during problem-solving tasks. Physiol. Behav. 209, 1–5. 10.1016/j.physbeh.2019.112609 31295452

[B5] Hsieh S. S. Fung D. Tsai H. Chang Y. K. Huang C. J. Hung T. M. (2018). Differences in working memory as a function of physical activity in children. Neuropsychology 32, 797–808. 10.1037/neu0000473 30124313

[B6] Kaliman P. Parrizas M. Lalanza J. F. Camins A. Escorihuela R. M. Pallas M. (2011). Neurophysiological and epigenetic effects of physical exercise on the aging process. Ageing Res. Rev. 10, 475–486. 10.1016/j.arr.2011.05.002 21624506

[B7] Valkenborghs S. R. Noetel M. Hillman C. Nilsson M. Smith J. Ortega F.. (2019). The impact of physical activity on brain structure and function in youth: a systematic review. Pediatrics 144, 1–14. 10.1542/peds.2018-4032 31554668

[B8] Villafaina S. Collado-Mateo D. Cano-Plasencia R. Gusi N. Fuentes J. P. (2019). Electroencephalographic response of chess players in decision-making processes under time pressure. Physiol. Behav. 198, 140–143. 10.1016/j.physbeh.2018.10.017 30389477

[B9] Villafaina S. Collado-Mateo D. Dominguez-Munoz F. J. Fuentes-Garcia J. P. Gusi N. (2018). Impact of adding a cognitive task while performing physical fitness tests in women with fibromyalgia A cross-sectional descriptive study. Medicine 97, 1–6. 10.1097/MD.0000000000013791 30572536 PMC6319791

[B10] Villafaina S. Collado-Mateo D. Dominguez-Munoz F. J. Gusi N. Fuentes-Garcia J. P. (2020). Effects of exergames on heart rate variability of women with fibromyalgia: A randomized controlled trial. Sci. Rep. 10, 1–8. 10.1038/s41598-020-61617-8 32198423 PMC7083950

[B11] Wunsch K. Meier M. Ueberholz L. Strahler J. Kasten N. (2019). Acute psychosocial stress and working memory performance: the potential of physical activity to modulate cognitive functions in children. Bmc Pediatrics 19, 1–15. 10.1186/s12887-019-1637-x 31382947 PMC6683391

